# Adjustment to disease and quality of life in people with vascular Ehlers-Danlos and Loeys-Dietz syndromes: A mixed-method study

**DOI:** 10.3389/fpsyg.2023.1019863

**Published:** 2023-02-28

**Authors:** Carolina Baeza-Velasco, Nuria Rodriguez, Laura Parra, Teresa Gutiérrez-Rosado

**Affiliations:** ^1^Laboratoire de Psychopathologie et Processus de Santé (LPPS UR4057), Université Paris Cité, Boulogne-Billancourt, Paris, France; ^2^Department of Emergency Psychiatry and Acute Care, CHU Montpellier, Montpellier, France; ^3^Institute of Functional Genomics, University of Montpellier, CNRS, INSERM, Montpellier, France; ^4^Health and Social Projects (with Heart), Barcelona, Spain; ^5^Department of Clinical and Health Psychology, University Autonomous of Barcelona, Barcelona, Spain

**Keywords:** Ehlers-Danlos sydromes, Loeys-Dietz syndrome, psychosocial adjustment, quality of life, anxiety, depression, rare diseases, mixed-method

## Abstract

**Background:**

Vascular Ehlers-Danlos (vEDS) and Loeys-Dietz syndromes (LDS) are hereditary disorders of connective tissue having severe vascular complications (HDCTv) which lead to an increased risk of premature death. Little is known about the impact of the disease in patient’s daily life.

**Method:**

Sixteen HDCTv patients (vEDS = 9 and LDS = 7), 16 age and sex-matched hypermobile Ehlers-Danlos syndrome patients (hEDS) and 18 healthy subjects (HS), responded to self-questionnaires assessing psychosocial adjustment, quality of life (QoL), anxiety, depression, pain, fatigue and sleep problems. Patients with HDCTv were also interviewed in order to explore qualitatively their experience with the disease.

**Results:**

Compared with HS, patients with HDCTv scored significantly higher on anxiety, depression, fatigue, sleep problems, and lower on QoL. Most HDCTv patients (93.8%) have optimal psychosocial adjustment. In addition, HDCTv patients scored higher on QoL and psychosocial adjustment, but lower in pain, fatigue, sleep problems, and depressive symptoms than hEDS patients. Four main themes were identified in qualitative analyses: living with HDCTv, knowledge/ignorance of the disease, health behaviors/self-care and coping strategies.

**Conclusion:**

Our results suggest that despite the negative impact of HDCTv on the patients’ daily lives, overall, they present an optimal disease adjustment which points to appropriate coping strategies. More research in psychosocial aspects of people with these rare diseases are needed to confirm these results and better understand their needs.

## Introduction

The heritable disorders of connective tissue (HDCT) are a group of genetic diseases affecting the proteins of the connective tissue matrix such as collagens, elastins, fibrillins, and tenascins. Fragility of tissues is a common feature of HDCT for which the prognosis can range from benign to life threating. Among HDCT with life-threatening complications are vascular Ehlers-Danlos syndrome (vEDS, a.k.a. EDS type IV), and Loeys-Dietz syndrome (LDS). vEDS is caused by mutations in collagen, type III, alpha-1 (COL3A1) and its prevalence is estimated between 1:20,0000–50,000 (Omar et al., 2021). Concerning LDS, it is caused by mutations in genes encoding for components of the transforming growth factor beta (TGFβ) signaling pathway: TGFBR1, TGFBR2, SMAD2, SMAD3, TGFB2, and TGFB3 (Velchev et al., 2021). As vEDS, LDS is a rare disease but its prevalence remains unknown (Gouda et al., 2022).

vEDS and LDS are part of HDCT also belonging to the group of hereditary thoracic aortic aneurysms and dissections (Fusco et al., 2022). Indeed, both conditions present multi-systemic manifestations (i.e., cardiovascular, musculoskeletal, craniofacial, ocular, and cutaneous; Velvin et al., 2019). Nevertheless, the most serious are those related to the cardiovascular system such as risk of aneurism and dissection of the aorta and other arteries (Velvin et al., 2019). vEDS and LDS can also present spontaneous organ rupture (e.g., spleen, intestine, and uterus; Chu et al., 2014; Frise et al., 2017; Frank et al., 2019).

Because of the exceptional arterial and organ fragility, life expectancy has been estimated at 51 years in vEDS (Frank et al., 2019), and 37 years in LDS (Loeys et al., 2006). However, as Pyeritz (2014) stated, advances in last decades concerning diagnosis, follow-up and treatment including surgery in LDS decreased acute dissection events and therefore increased life expectancy.

People affected by these HDCT with vascular complications (HDCTv) may being unaware they are affected until the first complication appears (Chu et al., 2014). These complications may require emergency interventions with an increased risk of subsequent morbidity and loss of physical function (Velvin et al., 2019). Thus, the diagnosis of a life-threatening disease such as HDCTv can lead patients to significant life changes in order to adapt and cope with uncertainty (de Ridder et al., 2008).

The psychosocial adjustment to illness, which is defined as the process of adjusting the internal needs to the new external demands given by the new life situation (Livneh and Antonak, 1997), is important in maintaining optimal quality of life (McCathie et al., 2002). Sub-optimal psychosocial adjustment may inhibit the implementation of coping strategies and adherence to medical recommendations, which in turn contributes to potential fatal risk (Connors et al., 2012). In this sense, exploring the experiential aspects of patients with serious illness is necessary to identify issues such as anxiety, depression, loss of will to live, as well as to predict positive health outcomes and understand complex decision-making processes (Schaufel et al., 2011).

Unfortunately, for HDCTv such as vEDS and LDS, works on psychosocial aspects are extremely rare (Velvin et al., 2019). A qualitative study by Connors et al. (Omar et al., 2021) explored adjustment and coping mechanisms in individuals with genetic aortic disease (*n* = 21) including LDS, Marfan syndrome and other conditions in the category of unspecified thoracic aortic aneurysm and dissection. Results of this study showed that patients may experience psychosocial distress and coping difficulties, even years after diagnosis. In addition, although the study by Johansen et al. (2021) did not investigate adjustment, this explored life satisfaction in patients with vEDS and LDS (*n* = 52). Results showed that the areas with a major proportion of participants satisfied were family life (69%), activities of daily living (69%), and relationships with their partners (68%). In contrast, the areas of somatic health and vocations were those with a less proportion of participants satisfied (<33%).

Particularly on Ehlers-Danlos syndromes, quantitative and qualitative studies on quality of life (QoL) and the influence of the disease in those affected exist. However, these studies are focalized either in the hypermobile subtype (hEDS) which is the most frequent form of Ehlers-Danlos syndrome among 13 subtypes, or these studied samples mixing different subtypes (e.g. Berglund et al., 2000, 2015; Bovet et al., 2016; Kalisch et al., 2019; Palomo-Toucedo et al., 2020). Thus, the specificities of vEDS concerning psychosocial adjustment remain poorly known, as well as those of people with LDS.

The objectives of this cross-sectional study with a mixed methodology (quantitative and qualitative) are firstly, to explore the psychosocial adjustment to disease and certain dimensions of QoL (physical functioning, vitality and general health) in people with HDCTv (vEDS and LDS), and compare them to two populations: healthy subjects (HS), and patients with hypermobile Ehlers-Danlos syndrome (hEDS) which belong to the same family of diseases (HDCT) but without life-threating complications. Secondly, we explored the life experience of patients with HDCTv through semi-structured interviews.

## Method

### Design and participants

This is a cross-sectional study with a mixed methodology (quantitative and qualitative), carried out in Spain and focused on people suffering from HDCTv (i.e., vEDS and LDS). The latter are patients with genetically confirmed vEDS and LDS recruited through the Spanish National Patient Association called *Asociación Nacional del Síndrome de Ehlers-Danlos e Hiperlaxitud* (ANSEDH). For comparison purposes, patients with hEDS and HS were also recruited through the same association and community, respectively. The participants were men and women, aged 18 years or older and native Spanish speakers. Exclusion criteria for HS were the presence of a chronic somatic disease or chronic pain.

### Instruments

A questionnaire collecting socio-demographic and health data was used. It included questions on age, height, weight, education level, employment and marital status, diagnosis (vEDS, hEDS, or LDS), age of onset of symptoms and age at diagnosis.

Usual fatigue, pain, and sleep problems (during the last 15 days) were measured using a verbal rating scale ranging from 0 (absent) to 4 (very intense).

Adjustment to illness was measured using the Psychosocial Adjustment to Illness Scale (PAIS; Derogatis, 1986). This instrument is composed of 46 questions divided into seven sections: social environment, extended family relationships, sexual relationships, domestic environment, professional domain, health care orientation, and psychological distress. The answers are formulated in the form of Likert scales. For all subscales, the lower the score, the better the fit. As the PAIS has not been previously used in a sample of HDCT, the internal coherence of the questionnaire was checked. The Cronbach’s alpha observed is 0.98.

Three areas of QoL were assessed using the 36-Item Short Form Health Survey (SF-36; Mchorney et al., 1993): “Physical functioning” which is considered an optimal indicator of mobility disability (Syddall et al., 2009) and has been extensively linked to the “Activities and Participations” component of the International Classification of Functioning, Disability and Health (Cieza et al., 2002). “Vitality” which assess energy and fatigue, and “General health perceptions.” Items of these subscales (10 items, 4 and 5 respectively) propose a hierarchical range of difficulties with a response format of Likert-Scale.

Anxiety and depressive symptomatology were assessed with the Hospital Anxiety and Depression Scale self-questionnaire (HADS; Zigmond and Snaith, 1983). The HADS consists of 14 items divided into two subscales, one assessing depression and the other anxiety. Each item corresponds to four response modalities rated from 0 (strong disagreement) to 4 (strong agreement). A higher score indicates a more severe symptomatology. For each of the two subscales the maximum score is 21, equal or higher than 11 points meaning clinically significant depressive symptomatology/anxiety.

For the qualitative component, a semi-structured interview guide was prepared based on literature and knowledge of the population by one member of the team. The guide was refined as necessary during data collection (Table 1). Due to the exploratory nature of the study, the questions were kept broad to allow participants to share their experiences.

**Table 1:** Interview guide.

What does the vEDS/LDS mean to you?
What would you say your life is like with this disease?
Do you think the disease has changed anything in you? – If so, can you explain what changes you are referring to?
What are the main concerns or difficulties you are currently experiencing?
How do you deal with these concerns or difficulties? What strategies do you use?
How do you see the future?
In your experience, do you think there are positive aspects associated with the disease?
Have you noticed any changes in the people around you since you were diagnosed? In the way they treat you, for example.
Of all the things you've told me, what do you think is the most important thing or the most important thing to remember?

### Procedure

First a call for participation in the study was sent by the patients association ANSEDH to its members with HDCTv. Once this group was recruited, the patient’s association contacted members with hEDS according to age range and sex of the HDCTv group (i.e., for matching purpose) to proposed them the study.

All patients (i.e., HDCTv and hEDS) who agreed to participate received an information note, informed consent and the self-questionnaires in word documents for the study by email. Once completed, they returned the documents by email.

HC were subjects who declared themselves free of diseases and in good physical health. They were recruited from the community and social networking services (e.g., Facebook), and matched by age and sex with the HDCTv group. They received, completed and returned the consent and self-questionnaire in the same way as patients.

Data collection for the quantitative part of the study took place between June and December 2019. In order to deep understand results of quantitative data, the research team decided to perform the qualitative part of the study. This was possible since all patients with HDCTv responded favorably to the question “Would you be willing to collaborate in another investigation in the future?” contained in the questionnaire of the quantitative study. Thus, in September 2020, the participants of the HDCTv group were recontacted by email or telephone to propose them the participation in the qualitative part of the study. Those who accepted signed a consent form which was a word document sent by email.

Due to the health crisis (COVID-19) and the geographical distribution of the participants, the interviews were conducted by telephone by two clinical psychologists (LP and CBV). The dates and times of the interviews were chosen according to each person’s availability. The interviews were audio recorded, transcribed verbatim and analyzed. The collection of qualitative data was done between September and November 2020.

### Analyses

*Quantitative:* Descriptive analyses were performed (percentages, means, standard deviation), binary logistic regression, Student *t*-test or Mann–Whitney *U* test (for continuous variables) and chi square test (for categorial variables) if binary logistic regression was not possible. The level of significance was set on.05 for all analysis. The quantitative data were analyzed using IBM SPSS version 22 statistical software.

Before to proceed with groups comparisons (i.e., HDCTv vs. HS, and HDCTv vs. hEDS) we performed intergroup comparisons on all the study variables between vEDS (*n* = 9) and LDS (*n* = 7) which compose the HDCTv group. No significant differences were observed in sociodemographic and health variables (Supplementary Table S1) which is concordant with results of Johansen et al. (2019).

*Qualitative:* The data were processed manually using inductive thematic analysis. This method proves to be very flexible and seems suitable for an exploratory study (Braun and Clarke, 2013). The steps followed for analyze included detailed readings of the interviews and coding process by each researcher separately, comparison of individual lists of categories identified, and refinement and agreement on a set of categories.

Three researchers participated in this process: the first author who is a clinical psychologist with an expertise in the study of psychosocial aspects associated with HDCT, mainly hEDS. The other two researchers (TGR and NR, clinical psychologist and nurse/anthropologist respectively) have experience in qualitative research but no prior knowledge concerning the diseases studied at the time of analyses and did not participate in the data collection. Triangulation defined as “the use of multiple methods or data sources in qualitative research to develop a comprehensive understanding of phenomena” (Patton, 1999), was thus ensured, on the one hand, by the complementarity of the quantitative and qualitative approach, and, on the other hand, by the joint analysis of three researchers. We report the study according to the Consolidated Criteria for Reporting Qualitative Research (COREQ statement; Supplementary Table S2).

## Results

### Quantitative results

From 20 patients with HDCTv contacted by the patient’s association, 16 agreed to participate in the study (response rate: 80%). Thus, vEDS (*n* = 9) together with LDS (*n* = 7; subtype 1 = 1; subtype 2 = 5; subtype 3 = 1) compose the HDCTv group. Patients with HDCTv were mainly females (13 women and 3 men) and are on average 39 years old (ET = 11.7). Ten patients (62.5%) have antecedents of HDCT in the family and six (37.5%) have lost a family member due to the illness. Ten patients reported having suffered at least one symptomatic event (arterial, gastrointestinal or respiratory).

Concerning the hEDS group, from 26 patients contacted by the patient’s association, 18 participated in this study (response rate: 69.2%).

### Comparison between HDCTv and HS

The comparison between HDCTv (*n* = 16) and HS (*n* = 18) showed that these groups are similar in all the sociodemographic variables assessed (Table 2). Concerning health variables, HDCTv patients scored significantly higher on usual fatigue and usual sleep problems than HS. In addition, participants with HDCTv have significantly lower scores on physical functioning, vitality and general health but higher scores on anxiety and depression than HS. However, from a categorial point of view, none participant from the HDCTv and HS group have scores exceeding the cutoff suggesting clinically significant levels of depression (HADS score ≥11). No differences were found between HDCTv and HS in terms of categorical anxiety, since four patients with HDCTv (25%) and one with HS (5.6%) exceeded the cut-off point suggesting clinically significant levels of anxiety (*p* = 0.152).

**Table 2:** Comparison of the study variables between HDCTv and HS.

	HDCTv	hEDS	OR (95% CI)	*p*
	*N* = 16 Mean (SD)/% [*n*]	*N* = 18 Mean (SD)/% [*n*]		
***Sociodemographics***
Age	39.0 (11.7)	38.9 (6.77)	0.999 (0.929–1.075)	NS
Sex				
Women	81.3% [13]	83.3% [15]	1.154 (0.198–6.735)	NS
With partner	81.3% [13]	72.2% [13]	0.600 (0.118–3.046)	NS
With children	43.8% [7]	55.6% [10]	1.607 (0.414–6.240)	NS
University studies	75% [12]	94.4% [17]	5.667 (0.561–57.234)	NS
***Health and psychosocial data***	***p*** *
Body mass index	21.9 (2.6)	23.4 (2.6)	1.277 (0.937–1.740)	NS
Usual pain intensity	1.06 (1.0)	0.61 (1.0)	0.648 (0.324–1.296)	NS
Usual fatigue intensity	1.38 (1.2)	0.50 (0.70)	0.399 (0.175–0.909)	0.029
Usual sleep problems	1.63 (1.3)	0.50 (0.70)	0.313 (0.124–0.791)	0.014
QoL dimensions
Physical functioning	68.7 (24.3)	97.7 (3.5)	–	<0.001*
Vitality	50.9 (13.5)	63.3 (12.8)	1.076 (1.012–1.144)	0.019
General health	41.0 (13.2)	77.7 (12.2)	1.316 (1.016–1.705)	0.037
Anxiety score (HADS)	8.1 (3.0)	4.1 (3.2)	0.674 (0.510–0.890)	0.006
Depression score (HADS)	3.3 (2.4)	1.2 (2.1)	0.620 (0.405–0.949)	0.028

HDCTv, hereditary disorders of connective tissue with vascular complications; HS, healthy subjects; SD, standard deviation; QoL, quality of life; HADS, Hospital Anxiety and Depression Scale; NS, non-significant.

* *U* Mann–Whitney test.

### Comparison between HDCTv and hEDS

Concerning sociodemographic data, only marital status is different between HDCTv and hEDS. Indeed, a greater proportion of people with HDCTv have a partner compared with hEDS (81.3% vs. 43.8%; *p* = 0.035). With respect to clinical and psychosocial data, binary logistic regression analyses controlled for marital status showed that the HDCTv group scored significantly lower on usual pain and fatigue intensity as well as in sleep problems and depression than the hEDS group. Moreover, in the three dimensions of quality of life assessed (i.e., physical functioning, vitality, and general health) and in all dimensions of adjustment to disease the HDCTv group obtained significantly better scores than the hEDS group. Indeed, the vast majority of patients with HDCTv present an optimal psychosocial adjustment to disease (93.8%) unlike patients with hEDS, a minority of whom present an optimal adjustment (25%; *p* = 0.002). These results are summarized in Table 3.

**Table 3:** Comparison of the study variables between HDCTv and hEDS.

	HDCTv	hEDS	OR (95% CI)	*p*
	*N* = 16 Mean (SD)/% [*n*]	*N* = 16 Mean (SD)/% [*n*]		
***Sociodemographics***				
Age	39.0 (11.7)	38.1 (6.5)	0.990 (0.918–1.067)	NS
Sex				
Women	81.3% [13]	87.5% [14]	1.615 (0.232–11.263)	NS
With partner	81.3% [13]	43.8% [7]	0.179 (0.036–0.887)	0.035
With children	43.8% [7]	37.5% [6]	0.771 (0.188–3.173)	NS
University studies	75% [12]	50% [8]	0.333 (0.075–1.489)	NS
***Clinical and psychosocial data***				***p*** *
Body mass index	21.9 (2.6)	24.3 (5.3)	1.262 (0.966–1.649)	NS
Age at outset of first symptoms	18.6 (15.7)	12.2 (6.0)	0.949 (0.882–1.020)	NS
Age at diagnosis	34.0 (13.0)	33.6 (7.6)	0.986 (0.915–1.063)	NS
Diagnosis delay (years)	12.3 (13.2)	21.5 (10.5)	1.062 (0.988–1.143)	NS
Usual pain intensity	1.06 (1.0)	2.81 (0.65)	23.69 (1.63–343.60)	0.020
Usual fatigue intensity	1.38 (1.2)	2.88 (0.71)	3.951 (1.38–11.25)	0.010
Usual sleep problems	1.63 (1.3)	3.0 (0.89)	2.505 (1.132–5.543)	0.023
QoL dimensions:				
Physical functioning	68.7 (24.3)	39.0 (24.4)	0.955 (0.922–0.990)	0.013
Vitality	50.9 (13.5)	27.1 (11.8)	0.856 (0.768–0.954)	0.005
General health	41.0 (13.2)	21.3 (10.7)	0.867 (0.775–0.971)	0.013
Adjustment to disease				
PAIS total score	28.6 (15.7)	75.1 (16.1)	1.204 (1.019–1.434)	0.029
Good adjustment (total score <51)	93.8% [15]	25% [4]	43.85 (3.842–500.50)	0.002
Health care orientation	8.7 (3.1)	15.5 (3.4)	2.197 (1.259–3.836)	0.006
Professional domain	3.9 (3.8)	11.0 (3.7)	1.577 (1.138–2.185)	0.006
Domestic environment	3.8 (3.3)	13.7 (4.1)	1.799 (1.231–2.630)	0.002
Sexual relationships	3.3 (3.9)	8.0 (4.2)	1.284 (1.022–1.613)	0.032
Extended family relationships	0.5 (1.3)	6.1 (3.8)	3.112 (1.339–7.230)	0.008
Social environment	2.3 (2.7)	9.8 (4.8)	1.625 (1.157–2.280)	0.005
Psychological distress	5.9 (3.4)	11.5 (3.8)	1.592 (1.153–2.198)	0.005
Anxiety score (HADS)	8.1 (3.0)	10.7 (4.5)	1.192 (0.962–1.476)	NS
Depression score (HADS)	3.3 (2.4)	8.8 (3.7)	1.655 (1.170–2.341)	0.004

HDCTv, hereditary disorders of connective tissue with vascular complications; hEDS, hypermobile Ehlers-Danlos syndrome; SD, standard deviation; QoL, quality of life; PAIS, Psychosocial Adjustment to Illness Scale; HADS, Hospital Anxiety and Depression Scale; NS, non-significant.

* Binary logistic regression controlled for marital status (“with partner”).

In terms of categorical depression, both groups of patients are significantly different since none subjects with HDCTv but five with hEDS (31.3%) exceeded the cut off suggesting clinically significant levels of depression (*p* = 0.043). However, no difference was found in categorical anxiety between these groups (HDCTv = 25% vs. hEDS =50%; *p* = 0.273).

### Qualitative results

The 16 patients with HDCTv who participated in the quantitative study accepted the qualitative interview. The characteristics of participants are summarized in Table 4.

**Table 4:** HDCTv patient’s characteristics.

Participants	Gender	Age	HDCTv	Length of interview
P1	Female	44	LDS	20 min
P2	Female	36	vEDS	18 min
P3	Male	42	vEDS	45 min
P4	Female	38	LDS	18 min
P5	Female	47	LDS	23 min
P6	Female	29	vEDS	32 min
P7	Female	39	LDS	26 min
P8	Female	32	vEDS	24 min
P9	Female	35	vEDS	26 min
P10	Female	23	LDS	40 min
P11	Male	28	vEDS	25 min
P12	Female	39	vEDS	30 min
P13	Female	40	LDS	16 min
P14	Female	54	vEDS	33 min
P15	Female	42	LDS	15 min
P16	Male	74	vEDS	23 min

HDCTv, Hereditary disorders of connective tissue with vascular complications; LDS, Loeys-Dietz syndrome; vEDS, vascular Ehlers-Danlos syndrome; P, participant; min, minutes.

We present a brief description of the four major categories identified and the themes within the categories with illustrative quotes.

1. Living with HDCTv (functional and emotional aspects)

- Normality: Several testimonials (*n* = 11) described that outside of vascular episodes patients can lead a normal life, have a family, a job, etc. Overall, participants reported functionality in various aspects of their lives. Some precautions were highlight as necessary, such as avoiding certain sport and to prevent stress.

This is a disease that has no symptoms, it is discovered when the aorta ruptured. I had surgery to avoid this, so I could lead my normal life, I continue to work, I have three children … [P1]

I continue to do my normal life but I take precautions, I lead a quiet life [P2]

Those who have not had an episode (vascular) lead a normal life [P3]

- Fear and uncertainty: the majority of participants (*n* = 12) expressed feel worried or anxious about health problems that they may suffer at any moment. Dealing with uncertainty is a constant in their lives.

Sometimes when I feel a strong pain, I sleep with the phone next to me in case I have to call because something happened. Even feeling that I am a strong person, my reality is that I live in fear [P14]

Afraid that one of my arteries will be dissected or one of the carotid and paravertebral kinkings that I have will create a thrombus and I will suffer brain damage or death… if I feel my head hurts or feel palpitations or tachycardia, I think it may be something bad… [P2]

Nothing to worry about… except that I think about the future and I know I'm going to have more problems [P11]

- Negative emotions in and regarding family members: 11 patients evoked feelings of fear, worry with respect to state and quality of life of relatives who also have the disease, and/or perceiving the fear in their love ones about their own condition. Guilt is another emotion present especially concerning pregnancy and the risk of genetic disease in the offspring. In many cases, patients have faced the premature death of a family member due to the disease which increase fears in the family.

Psychologically, the only thing that annoys me is knowing that my nephews and my son have it too (…) I hope that nothing is going to happen to my son and that the diagnosis does not worsen… [P16]

I was considered doing an interruption of the pregnancy, but then we decided to go ahead. I already knew then that my eldest son had the disease, but what worried me was my youngest son, I had many feelings of guilt towards him because I was the one with the disease [P5]

My mother lives more alert and I suppose also in fear that something might happen to us… [P2]

1. Knowledge/ignorance of disease

- Diagnosis: The announcement of the diagnosis was often lived as a hard experience. However, for eight patients naming the disease and receiving information about it allowed them to take steps to prevent complications, understand and make sense of their early experience of feeling different or sick, as well as socially legitimize their condition.

Having the diagnosis reassures me about prevention, because my mother and brother died from the disease [P5]

I spent 30 years without knowing the diagnosis and something could have happened. But now with the care that I have, with the calmer life that I lead, the controls that I do, I see it more difficult for something to happen to me [P7]

Since I was diagnosed I no longer have to prove anything to anyone, and to be able to prove that I really couldn't work because my hands hurt, my elbows hurt … [P13]

- Health professionals: Four participants describe dealing frequently with the lack of knowledge and unpreparedness of health professionals for the management of their diseases.

There are doctors who don’t know about the disease, don’t even know how to write it… You have to see several doctors, the one who sees us annually doesn't even want to take a blood test for fear that something will happen, it’s exaggerated … In general, there is a lot of ignorance in the medical world about the vEDS [P2]

Doctors hardly study this disease at university. It is a rare disease and they waste time learning about it, so little is known. Sometimes I prefer not to go to medical appointments because they don't know what it is, we know more than them… That's why we go to congresses, there we look for doctors for their ask questions [P3]

1. Heath behaviors/self-care

- Medical adherence: Patients are very aware of the need to monitor their health and follow the recommendations of doctors to prevent complications. This optimal medical adherence/compliance, which appear in data of eight patients, allow them to increase their feelings of control over events and self-efficacy.

I try to be very strict with medical check-ups, I always try to carry it as naturally as possible. But I am very strict with the controls [P13]

The truth is that any day something can happen to you, that's why I go to all my check-ups and I try to have good trust with the doctors because I know that one day they will be there in case something happens to me one day and I try to do everything they tell me [P6]

- Modulation of lifestyle: In order to minimize injuries and complications, several patients (*n* = 10) have introduced behavioral changes to their routines such as healthier eating, exercises to get fit and reduce stress, changing jobs if considered stressful, etc.

I started to take better care of myself, I didn't play sports and I started doing Pilates, watching what I eat … [P1]

Now I am calmer and more cautious. I also try to take better care of my diet, to eat healthier and to exercise in a way moderate. I have become more homely [P2]

Before, I would never have gone on a diet; now I seek health … I do yoga, I would never have done that before. I am looking for activities that calm down, I walk, I exercise to be calmer … [P3]

1. Coping strategies

- Positive reappraisal: 12 patients were able to find positive sides to the context of the illness or create positive meanings by focusing on personal growth, acquired skills, identification of values and reprioritization.

I was very lucky because I was detected the disease early … I see my brothers (also sick) and other people who are very bad, so I think I am lucky … [P1]

I have learned that I can handle things that I thought I couldn't, I have learned to listen to myself, to relax and calm my anxiety, although I don't always succeed … to be calmer, to enjoy my home more, the tranquility, the little things … [P2]

At work I was very involved, it was the most important, there comes a time when it is no longer but the family. Priorities change, I enjoy other things, seeing friends … Now I’m closer to my mother, my sister … [P3]

- Humor and optimism: Six patients consider reported that having a positive cognitive approach is helpful in dealing with concerns. They describe using strategies such as humor and optimistic thoughts to build resilience.

I take it with a lot of humor. What I have realized is that those who are worse off are usually the ones who make the most jokes. I know that most people I have met take it with a lot of jokes and I love that [P11].

I try to think that I am fine and that everything will be fine. I try to think that I can die at 90, without anything happening of that could happen to me at the vascular level [P2]

- Cognitive avoidance: Eight patients show avoid unpleasant or distressing *thoughts about disease. Efforts to distract from* negative thoughts were described.

I try not to think too much, because if I think, and think about things a lot, it's bad. So I try to live every day, I work 8 hours so I live busy from top to bottom … [P6]

I try not to think too much because if the day doesn't eat you up, negative thoughts eat you up and I don't want that [P13]

- Focus on present: to think in the future is often anxiogenic for patients. Thus, to focus on present is a strategy frequently used by eight patients. Plans are often thought only in the short term since seem more realistic and emotionally tolerable.

Honestly, I live day to day. Before I was not so aware, because a young person plans all the time without thinking that at some point this might end, or that you are going to end up in a hospital [P7]

Since I have the diagnosis I do not see myself, I do not visualize myself much further, I try to live now, I do not visualize myself when my son is older, the truth is that I do not visualize it. I try to live in the now, to give thanks when I get up in the morning, what I do and what I want is to enjoy my family as long as possible [P13]

- Acceptance: Six patients declared have accepted their condition. They recognized this aspect as a key of the process of “normalize” the day-to-day life. In addition, acceptance allows them to orientate the focus on aspects of life on which they may have more control.

Life is like that, recalculate the route and continue… there is no other option … I realize that definitely nothing is in my hands, the only thing I can do is go to the doctor and check myself [P7]

I was born like this and I cannot erase it from the DNA, but what I can do as I have been until now is to continue taking it calmly, with humor, continue living my life with the greatest possible normality [P10]

- Family and friend support: Several testimonials (*n* = 10) identified the support of relatives and friends on a day-to-day basis as well as during medical visits as a very important help to lead with difficulties and negative emotions.

I would not have been able to overcome even half of things without her (mother) [P10]

I have support from them (family), my brother is also sick and he’s more seriously ill since he has had significant aortic problems. My father died for this. We support each other … [P2]

## Discussion

This study aimed to explore the psychosocial adjustment to disease and certain dimensions of QoL in people with HDCTv (vEDS and LDS) through a mixed methodological approach.

As expected, the quantitative results showed that compared to HS, people with HDCTv have lower QoL, specifically in the areas of physical functioning, vitality and perception of general health. In addition, they had more usual fatigue and sleep problems as well as higher levels of anxiety and depression symptomatology compared with HS. Indeed, it is well known that chronic diseases, especially life-threatening ones, have a negative impact on the QoL of those affected (Velvin et al., 2019). Though, no differences were observed between patients with HDCTv and HC concerning pain intensity (both groups present low usual pain intensity). This contrast with the results of Johansen et al. (2019) who found a high proportion (79%) of patients with vEDS and LDS suffering from chronic pain. Plausibly, these differences may be related to pain assessment. In the study of Johansen et al. (2019) assessment consisted in one item (yes/non) asking for history of chronic musculoskeletal pain for a period of >3 months last year, while in our study the measure was focalized in the usual pain intensity experienced during the last 15 days. Thus, we cannot exclude that patients of our sample had passed a chronic pain period.

Concerning the comparison between HDCTv and hEDS (the last also belonging to the HDCT but without life-threating complications), patients with HDCTv have a better QoL in the three dimensions assessed as well as better psychosocial adjustment to disease than hEDS. Indeed, the vast majority of participants with HDCTv (93.8%) scored in the optimal adjustment range, which contrasts with the scores of patients with hEDS, who mostly (75%) show poor adjustment.

At first glance, these results may be surprising since HDCTv are serious diseases in which those affected live with the threat of severe vascular events without warning, and having a live expectancy between 31 and 51 years old (Loeys et al., 2006; Frank et al., 2019). hEDS in contrast, is a HDCT and the subtype of EDS considered as the least severe (Levy, 2004). Nevertheless, although hEDS is not life-threatening like HDCTv, its clinical picture is characterized by a variety and accumulation of symptoms of long duration that make hEDS a highly disabling condition (Baeza-Velasco et al., 2018). Indeed, studies show that patients with hEDS have severe functional impairment determined mainly by pain and fatigue (Voermans and Knoop, 2011; Kalisch et al., 2019). In this sense, our results show that people with HDCTv present significantly less depression, pain, fatigue and sleep problems compared with people with hEDS. This may help explain the differences obtained in QoL between these two groups of patients.

In a similar line, Johansen et al. (2021) who studied life satisfaction in patients with HDCTv, reported that a high proportion of these patients (≥68%) were satisfied with life specifically with activities of daily living, family life and relationships with their partners. In addition, they observed that low levels of fatigue, anxiety and multi-organ burden were associated with higher scores on life satisfaction in several domains.

Diagnosis is another clue to understand the differences observed on adjustment to disease and QoL between both groups of patients. From a purely descriptive point of view, the diagnosis delay is 9 years higher in hEDS than in HDCTv. The HDCTv studied here (vEDS and LDS) can by molecularly verified which facilitate recognition. This is not the case of hEDS whose genetic abnormality is still not identified. Thus, hEDS is the least recognized HDCT mainly due to the lack of confirmatory laboratory/molecular tests (Castori, 2012). In this regard, the diagnosis of a chronic disease favor the implementation of coping strategies (Sacheti et al., 1997). On the contrary, a prolonged delay in diagnosis maintains the anxiety and waiting of the affected persons, which in turn delays adaptation (Besson et al., 2020).

Our qualitative results support these observations and shed some light on quantitative results. Narrative data show us that for many HDCTv patients, to receive the diagnosis was a hard experience but positive and even relieving. Indeed, having the name of the disease, patients were able to seek information, learned how prevent complications, make changes in their lifestyle and legitimize their health status socially. This could contribute to their good adjustment to the disease. In this sense, we observed that most patients with HDCTv adhere with medical recommendations showing a disciplined compliance with medical controls and changes to their habits to avoid complications (e.g., modulation of physical activity, diet). This is consistent with results of Connors et al. (2015), who also reported optimal medical adherence in patients with different aortopathies including HDCT such as LDS (*N* = 21).

Moreover, qualitative data show that most patients experience fear and uncertainty regarding the course of their disease and that of their family members who are also affected. However, patients’ strategies to cope with stress are varied and mostly functional (e.g., positive reappraisal, social support, acceptance, and humor). This is confirmed by the fact that despite the great stressors with which they must deal, only four participants with HDCTv present clinically significant anxiety and none have clinically significant depressive symptomatology. This is consistent with the results of Connors et al. (2015) who explored adjustment and coping mechanisms in individuals with genetic aortic disorders including LDS. They observed that dysfunctional coping strategies were infrequent among these patients and that they experienced minimal emotional distress. Speculatively, these optimal coping strategies may appear in these patients from good psychological resources at individual level and by learning. Indeed, many patients come from families leading with the disease from several generations. Thus, some strategies of coping could be observed and learnt from relatives.

The characteristics observed in HDCTv in this study are consistent with the literature on the individual factors associated with adjustment to illness (e.g., coping strategies; Dekker and de Groot, 2018). Other factors impacting adjustment are those related to the illness itself. In this sense, most of interviewees emphasize that apart from the vascular episode, the disease has no or few symptoms which allows them to lead a life they consider normal. In the current context where chronic diseases are on the increase, health is defined not as the absence of disease but rather as the ability to adapt and self-manage (Huber et al., 2011). In this sense, a satisfying life in many areas is possible for these patients.

A critical point emerged in interviews is the perception of the lack of knowledge of the disease on the part of health professionals. This is consistent with the results of one of the few qualitative studies carried out on patients with vEDS, which identified lack of knowledge of the disease in emergency departments as one of the most common sources of frustration (Shalhub et al., 2020). Thus, disseminate knowledge about HDCTv is needed to improve the diagnosis and management of these patients.

Finally, in view of the important differences in psychosocial aspects observed between HDCTv and hEDS patients, more efforts are needed in order to design studies aimed to elucidate the psychosocial specificities of different subtypes of HDCT rather than mixing subtypes as is often the case, especially with the Ehlers-Danlos syndromes. In this sense, and from a clinical perspective, these results should be considered for group interventions for patients with HDCT. Patients with different HDCT in the same group may not work optimally if there is heterogeneity regarding psychosocial functioning as observed in this study. In such a therapeutic context, patients may not feel understood or identified with the rest of the group and present very different needs, which may interfere with adherence to the intervention and in the group climate. In addition, the assessment of psychosocial adjustment and QoL should be integrated to the clinical follow-up of patients with HDCTv in order to detect difficulties beyond the somatic level and guide healthcare accordingly.

This study has several limitations. Concerning the quantitative component, the small sample size and the transversal design which does not allow inferences on the directionality of the associations. In addition, the recruitment through a patient association which implies that the generalization of results is compromised. In addition, although 8 out 16 participants with HDCTv sent us the document of their genetic test, the rest of patients self-reported their diagnosis. The HS status was also self-declared, therefore somatic health problems cannot be excluded in this group. Moreover, we do not have the data about treatment and medications which may impact overall physical factors, mental health and quality of life. It was not possible neither to examine differences in psychosocial results according to the vEDS variant (not reported in this study) and the LDS subtype due to the small sample size. These aspects should been considered in further research.

Concerning the qualitative part, data saturation was not reached and we were unable to interview more people with HDCTv since the convenience sample of HDCTv was limited in number (rare disease, adherents to an association). Thus, additional data is probably necessary to capture the experience of patients.

Despite these limitations, this study contributes with data that make an understudied clinical population more visible from a psychosocial point of view. Further research is needed to deepen existing knowledge about the burden of these diseases, as well as the strategies to facilitate psychosocial adjustment in those affected.

## Data availability statement

The data that support the findings of this study are available from the corresponding author upon reasonable request.

## Ethics statement

Ethical review and approval was not required for this study of human participants in accordance with the local legislation and institutional requirements. The patients/participants provided their written informed consent to participate in this study.

## Author contributions

CB-V designed the study, collected the quantitative data, and performed the statistical analysis. LP and CB-V conducted the semi-structured interviews. LP transcribed interviews. NR, TG-R, and CB-V analyzed qualitative data. CB-V drafted the manuscript. All authors contributed to the article and approved the submitted version.

## Conflict of interest

The authors declare that the research was conducted in the absence of any commercial or financial relationships that could be construed as a potential conflict of interest.

## Publisher’s note

All claims expressed in this article are solely those of the authors and do not necessarily represent those of their affiliated organizations, or those of the publisher, the editors and the reviewers. Any product that may be evaluated in this article, or claim that may be made by its manufacturer, is not guaranteed or endorsed by the publisher.
