# Supplementary material for: Adjustment to disease and quality of life in people with vascular Ehlers-Danlos and Loeys-Dietz syndromes: A mixed-method study
Source: Front Psychol. 2023 Feb 28;14:1019863. doi: 10.3389/fpsyg.2023.1019863 (PMC10011476; doi:10.3389/fpsyg.2023.1019863)
Supplement: Supplementary file 1 [file Table_1.docx]

Table S1. Descriptive data and comparison of the study variables between vEDS and LDS.

| ***Sociodemographics*** | **vEDS** | **LDS** | ***p**** |
| --- | --- | --- | --- |
|  | **n = 9**  **Mean (SD) / % [n]** | **n = 7**  **Mean (SD) / % [n]** |  |
| Age | 40.1 (14.5) | 37.5 (7.9) | .681 |
| Sex:  Women | 66.7% [6] | 100% [7] | .213 |
| With partner | 66.7% [6] | 100% [7] | .213 |
| With children | 44.4% [4] | 42.9% [3] | .951 |
| University studies | 66.7% [6] | 85.7% [6] | .585 |
| ***Health and psychosocial data*** |  |  | ***p*** |
| Family history of HDCT | 77.8% [7] | 42.9% [3] | .302 |
| Death in the family due to HDCTv | 44.4% [4] | 28.6% [2] | .633 |
| Symptomatic event^α^ | 55.6 [5] | 71.4% [5] | .724 |
| LDS subtype :  1  2  3 |  | 1  5  1 |  |
| Body mass index | 21.5 (2.0) | 22.5 (3.3) | .681 |
| Usual pain intensity | 1.1 (1.1) | 1.0 (1.0) | .918 |
| Usual fatigue intensity | 1.2 (1.4) | 1.5 (0.9) | .470 |
| Usual sleep problems | 1.8 (1.6) | 1.2 (0.7) | .606 |
| QoL dimensions:  Physical functioning  Vitality  General health | 63.3 (30.8)  50.0 (15.8)  41.1 (13.9) | 75.7 (10.5)  52.1 (11.1)  41.0 (13.4) | .758  .837  .955 |
| PAIS total score | 31.2 (17.9) | 25.4 (13.0) | .408 |
| Good adjustment (PAIS total score <51) | 88.9% [8] | 100% [7] | .378 |
| Anxiety score (HADS) | 8.4 (3.3) | 7.8 (2.7) | .758 |
| Depression score (HADS) | 3.7 (2.9) | 2.8 (1.7) | .606 |

HDCT= hereditary disorders of connective tissue with vascular complications. HDCTv = hereditary disorders of connective tissue with vascular complications. HS=healthy subjects. SD = standard deviation. QoL= quality of life. HADS= Hospital Anxiety and Depression Scale. *=U Mann Whitney test for continuous variables and chi square or Fisher’s exact test for categorial ones. ^α^ = arteria, gastrointestinal or respiratory.
